# Phosphatase and Tensin Homologue: Novel Regulation by Developmental Signaling

**DOI:** 10.1155/2015/282567

**Published:** 2015-08-03

**Authors:** Travis J. Jerde

**Affiliations:** Department of Pharmacology and Toxicology, Indiana University School of Medicine, IU-Melvin and Bren Simon Cancer Center, Indianapolis, IN 46202, USA

## Abstract

Phosphatase and tensin homologue (PTEN) is a critical cell endogenous inhibitor of phosphoinositide signaling in mammalian cells. PTEN dephosphorylates phosphoinositide trisphosphate (PIP_3_), and by so doing PTEN has the function of negative regulation of Akt, thereby inhibiting this key intracellular signal transduction pathway. In numerous cell types, PTEN loss-of-function mutations result in unopposed Akt signaling, producing numerous effects on cells. Numerous reports exist regarding mutations in PTEN leading to unregulated Akt and human disease, most notably cancer. However, less is commonly known about nonmutational regulation of PTEN. This review focuses on an emerging literature on the regulation of PTEN at the transcriptional, posttranscriptional, translational, and posttranslational levels. Specifically, a focus is placed on the role *developmental signaling pathways* play in PTEN regulation; this includes insulin-like growth factor, NOTCH, transforming growth factor, bone morphogenetic protein, wnt, and hedgehog signaling. The regulation of PTEN by developmental mediators affects critical biological processes including neuronal and organ development, stem cell maintenance, cell cycle regulation, inflammation, response to hypoxia, repair and recovery, and cell death and survival. Perturbations of PTEN regulation consequently lead to human diseases such as cancer, chronic inflammatory syndromes, developmental abnormalities, diabetes, and neurodegeneration.

## 1. Introduction

**Figure 1:**
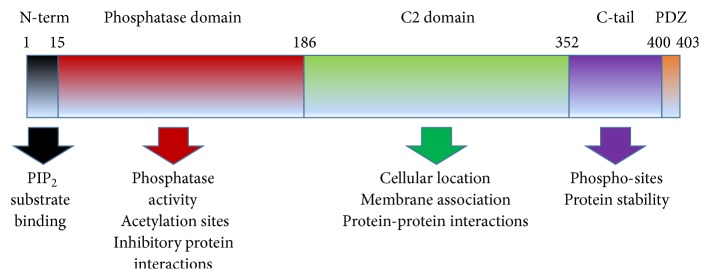
Protein domains of PTEN. PTEN has five distinct domains, consisting of an N-terminal PIP binding domain, the phosphatase domain responsible for its enzymatic activity and containing acetylation sites responsible for regulating this phosphatase activity, the regulatory C2 domain responsible for its cellular location and protein-protein interactions including those that modify enzyme activity or localization, the less understood C-tail containing phosphorylation sites thought to be critical for PTEN's stability, and finally the C-terminal PDZ domain.

Phosphatase and tensin homolog (PTEN) is a ubiquitously expressed protein that functions as a phosphatase to dephosphorylate phosphatidylinositol (3,4,5)-trisphosphate (PtdIns(3,4,5)P_3_ or PIP_3_) by catalyzing the dephosphorylation of the 3′ phosphate of the inositol ring in PIP_3_ [1]. The resulting product of this reaction is the biphosphate product PIP_2_ (PtdIns(4,5)P_2_). Since PIP_3_ is a primary activator of the signaling intermediate Akt, dephosphorylation of PIP_3_ by PTEN results in inhibition of the AKT signaling pathway. Akt is a serine/threonine-specific protein kinase that is critical for many cellular functions including cell proliferation, apoptosis, transcription, and cell migration and structure [1]. PTEN also functions as a protein phosphatase by dephosphorylating proteins including focal adhesion kinase in the cytosol and Erk, histone H1, RAD51, and CENC-P in the nucleus. Therefore, PTEN has effects on cell migration via integrin signaling, chromatin remodeling through its interactions with histones, cell cycle progression and arrest independent of Akt signaling, DNA repair via its modulation of RAD51, and centrosome stability via its role with CENC-P. PTEN's structure, described in Figure 1, consists of a phosphatase domain harboring the active site and enzymatic function of the protein and a C2 domain responsible for the phospholipid membrane binding site [2]. As such, the C2 domain is responsible for cellular location and allows PTEN localization to membrane-bound PIP_3_; this promotes PTEN's phosphoinositide phosphatase function by locating it to the cellular location of its substrates [2]. The functions of the C-terminal and PDZ domains are less defined [2].

Because the downstream signaling pathways that PTEN effects modulate very diverse and dynamic cellular functions, PTEN is responsible for regulating the signals of an abundance of mediators including cytokines, growth factors, integrins, and autacoid ligands of G-protein-coupled receptors [3–5]. The cellular mechanistic effects of PTEN function and intrinsic Akt inhibition include modulation of many pathways; this includes cell cycle progression via p27^Kip1^ [6], Wee1, p21, and cyclin D [7]; mTOR-mediated cellular signaling [8]; apoptosis and survival effects mediated by Bim [9], MDM2-p53 [10], Bcl-2, and Bax [11]; cellular structure regulation mediated by laminins and cytoskeletal protein expression [12]; glycogen and glucose regulation and glycolysis by glycogen synthase, 6-phosphofructo-2-kinase, and AS160 [13]. Adding to the broad activity of PTEN are the numerous examples of signaling crosstalk with other transduction pathways including the Erk, p38, and JNK mitogen-activated protein kinases [14], the TGF*β* and BMP-associated SMAD pathways [15], the cytokine-mediated Jak-STAT pathway [16], and IP_3_-mediated calcium signaling [17]. Additionally, interesting recent findings have demonstrated that PTEN modulates the DNA damage response and repair via its inhibition of Akt [18]. Because of this diversity, any mediators that modulate PTEN expression levels or function have substantial effects on cellular function.

As a critical cellular protein that modulates numerous processes, it is expected that PTEN plays a major role in regulating proper development. Yet, PTEN is still primarily known as a tumor suppressor—the loss of function of which leads to tumorigenesis. While this may be true, the last decade has seen a greatly enhanced understanding of how PTEN is regulated, particularly as this relates normal growth and development. This review takes a critical look at the literature focusing on PTEN regulation, the effects of this regulation during mammalian development, and how it is regulated by classical developmental signal transduction mechanisms.

## 2. PTEN in Developmental Biology

### 2.1. Early Embryonic Development

PTEN is highly and ubiquitously expressed throughout developing embryos. It is expressed as early as the embryonic stem cell stage, being detectable in embryonic day 3.5 blastocysts in both extraembryonic and embryonic tissues [19]. PTEN is required for critical hallmarks of developmental biology including cell proliferation, growth, death, and migration. In many tissues, PTEN expression coincides with the need to repress or induce proliferation or differentiation in cells, depending on the tissue need in each location and time point.

**Figure 2:**
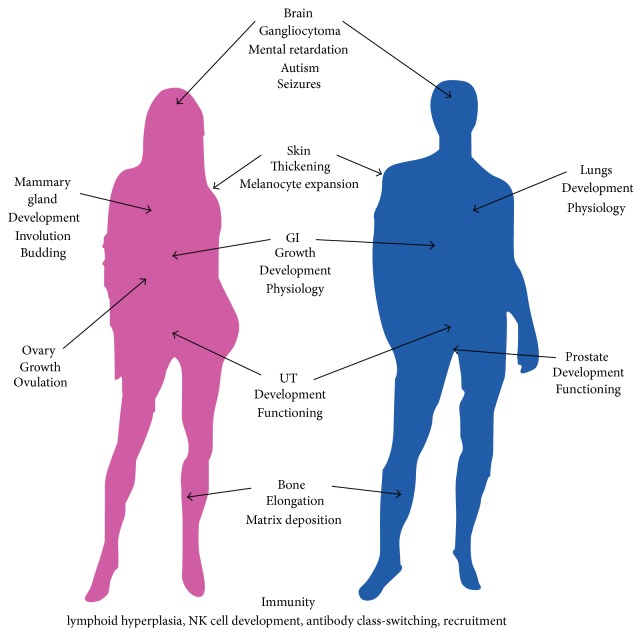
Precise regulation of PTEN expression, activity, or localization has profound effects on the development of numerous organ systems. Disruption of PTEN at the level of transcription, mRNA stability, protein stability, enzymatic function, or cellular location results in disruption of these developmental systems.

At an organismal level, PTEN plays several critical roles in the early control of the patterning of embryos. First, PTEN regulation of cell migration is known to control specification of the anterior-posterior axis of mouse embryos [20]. Embryos that lack PTEN expression exhibit a notable set of morphological defects including the failure to correctly specify the anterior-posterior body axis, improper separation of body foci, and decreased coordinated migration of cells along the body axis. PTEN is also necessary for gastrulation of embryos. PTEN is highly expressed from the midblastula transition through completion of gastrulation and embryonic layer separation [21]. In this process, PTEN coordinates cell cycle elongation and cellular morphogenesis. Embryos lacking PTEN fail to exhibit proper patterning from the blastula into the germ layers. Lethality of these embryos soon follows. At the postgastrulation stage, PTEN localization can be found throughout ectodermal, endodermal, and mesodermal-derived tissue, including the developmental precursors of the central nervous system and skin, the liver and gastrointestinal tract, the urogenital sinus, heart, and skeletal muscle [19, 20]. Anatomy of PTEN systems effects highlighed in Figure 2. Following the separation of the germ layers, PTEN exerts dynamic roles throughout development, across numerous organ systems. The following highlights recent findings.

### 2.2. Nervous System Development

The high prevalence of germline PTEN mutations in Cowden disease and Bannayan-Riley-Ruvalcaba syndrome, Lhermitte-Duclos syndrome, autism spectrum disorders, and CNS axon regeneration demonstrates the significance of PTEN in central nervous system development and physiology. The brain has been the most extensively studied organ in the area of PTEN's role in developmental biology [22], primarily because of its known role in the development of gliomas and medulloblastomas. Identified syndromes associated with germline PTEN in the CNS include Lhermitte-Duclos disease [23], dysplastic gangliocytoma [24], gray matter heterotopias [25], mental retardation [26], autism [27], and seizures [28].

Cell type-specific mutations of PTEN in animal models demonstrate developmental phenotypes and resulting pathology. Glial cell conditional null mutation of the Pten gene promotes seizures and ataxia early in life in mice and promotes premature death [28]. Brain size in these mice increases progressively over time relative to wild-type littermates, and these mutants develop hydrocephalus and untoward lateral ventricle growth [28]. These animals exhibit neural dysplasia, thickened internal granule cell layers, and a progressive increase in neuronal soma size [28]. Further, mice with oligodendrocyte-specific PTEN deletion exhibit disruption in the myelination of axons [29]. Importantly, these murine effects are similar to many common features associated with PTEN mutations in the human CNS [30].

PTEN deletions in terminally differentiated neurons result in a number of striking phenotypes. Neuronal-specific PTEN deletion promotes soma hypertrophy, macroencephaly, and increased axonal outgrowth. This latest finding has led to the hypothesis that PTEN inhibition may be a potential therapeutic mechanism for inducing axonal outgrowth in spinal injury patients [31]. These mice also exhibit increased dendritic spine density, increased caliber of neuronal projections, and alterations in synaptic transmission [31]. Neurons from these mice exhibit disruption in Bergmann-glia connections and disorganized cerebellar architecture [31]. Neuronal-conditional PTEN null mice also display hyperactivity, signs of anxiety, heightened response to excitatory stimuli, and disrupted social interaction [32]. Findings from these studies have prompted the hypothesis that PTEN expression anomalies may be at least in part responsible for CNS-related disorders in humans.

### 2.3. Dermal Development

PTEN-mediated signaling has long been known to be critical for the cytoprotection of both epithelial cells and melanocytes in the skin. Interestingly, PTEN has recently been shown to be responsible for regulating the events and timing of epidermal development [19]. This may have been predictable given the incidence of benign tumors of the skin in Cowden's syndrome, a known PTEN-driven condition. Conditional PTEN deletion induces hyperplasia of squamous cells resulting in mounted skin dermis and papillomas [33]. Additionally, mice with specific melanocytic PTEN deletion exhibit increased melanocyte expansion [34].

### 2.4. Hematopoietic Development

PTEN is necessary for proper development, lineage fate, and cell function in T cells, B cells, myeloid cells, NKT cells, and hematopoietic stem cells (HSCs), as demonstrated by several elegant studies involving global heterozygous and cell-conditional null PTEN models [35–40]. It has been known that PTEN (+/−) mice exhibit lymphoid hyperplasia progressing to T-cell lymphoma complete with large nodal masses [36], and it has now also been reported that PTEN (+/−) mice develop autoimmune disorders at an early age due to abrogated PTEN function [37]. Cell-type T-cell conditional PTEN null mice exhibit lymphadenopathy, splenomegaly, and hyperplastic enlarged thymus. Similar to the global PTEN heterozygous mice, conditional nulls also exhibit autoreactivity evidenced by increased levels of autoantibodies, expanded T-cell proliferation, and increased cytokine synthesis and release [38].

Indirect studies show that PTEN-mediated signaling plays a role in natural killer (NK) cells. Unfortunately, true cell-selective knockout of PTEN in natural killer (NK) cells has not been reported due to the lack of a cell-type specific promoter. However, purified NK cells from PTEN immune-system null mice exhibit abrogated maturation and activation [39]. Studies have demonstrated that when these cells were adoptively transferred into NK null mice harboring metastatic melanoma cells, the null cells exhibit strongly decreased protection from metastases relative to wild-type NKT cells [39, 40]. These results demonstrate that one critical role of NK cells, namely, tumor targeting, is attenuated in PTEN null NK cells. In addition, B-cell lineage-specific PTEN null mice exhibit expanded splenocytes, mesenteric lymph node cells, and peritoneal cells [41]. B cells deficient in PTEN display enhanced proliferation upon epitope or mitogen stimulation, and defective class-switch antibody recombination resulting in decreased production of IgA and IgG antibodies and elevated IgM production [41].

Neutrophils isolated from mice with PTEN-null myeloid lineage cells display increased actin polymerization with enhanced membrane ruffling, pseudopodia formation, chemotaxis, and migratory speed [42]. Inflammation in these mice is associated with vastly increased neutrophil recruitment [42]. Paradoxically, these mice are more susceptible to infection with* Leishmania* and exhibit a slower clearance of infection than wild-type littermates. This disconnect appears to be due to decreased secretion of tumor necrosis factor (TNF) by mutant macrophages in these mice [43]. To date, little else is known about the development of monocytes in myeloid-specific PTEN null bearing animals.

Deletion of PTEN in murine hematopoietic stem cells in mice results in a very rapid onset of myeloproliferative disorders and an eventual depletion of the stem cell pool, which, despite their proliferative nature, results in the loss of the ability of these stem cells to produce all lineages needed for proper immune functioning [44]. These myeloproliferative disorders progressed to acute myeloid leukemia or acute lymphoblastic leukemia [44]. In a demonstration that Akt-signaling is downstream of this phenotype, increased HSC proliferation and even the development of myeloproliferative disorders and leukemia are all rescued when the mice were treated with rapamycin [44].

### 2.5. Visceral Organ Development

There are numerous studies to date examining the developmental effects of organ-specific PTEN mutation in mouse models. This review summarizes the highlights of these studies here. Mammary ducts in tissue-specific conditional PTEN null mice exhibit loss of differentiation, enhanced growth, excessive side branching, and precocious budding relative to wild-type controls [45]. These phenotypes are reported during puberty but also manifest their effects during mammary expansion during pregnancy. Notably, mammary gland involution after pregnancy was also defective [45]. PTEN is important for the normal physiology of oocytes, as PTEN deficiency in murine oocytes causes the entire oocyte pool to become activated prematurely [46]. This proves to be critical for fertility and reproduction, as premature oocyte activation resulted in females having a maximum of one normal-sized litter before they became infertile at 12-13 weeks of age [46].

FabpCre-(Ptenflox/flox) mice exhibit a number of interesting phenotypes in the genitourinary system. PTEN deletion occurs in the bladder, ureter, kidney, colon, prostate, seminal vesicles, and vagina of these mutants [47]. Epithelial hyperplasia was omnipresent in these animals. As expected, deletion of PTEN resulted in increased susceptibility to chemically induced carcinogenesis [48]. Similarly, epithelial hyperplasia is present in organ specific PTEN knockouts, followed by intraepithelial neoplasia and ultimately by invasion of the cancer cells as invasive adenocarcinoma [49–51]. Embryonic deletion of PTEN in the lower urinary system results in a loss of proper differentiation within affected cells [52], ultimately resulting in loss of proper patterning and growth control. Based on these data and the collection of phenotypes observed in PTEN deleted cells and animals, it is likely that a loss of differentiation of cells plays a master role in PTEN's role in organ development.

Recent reports have demonstrated a critical role for PTEN in the branching morphogenesis and nephron patterning of developing kidneys. PTEN has been shown to be indispensable for developing bud outgrowth, mesenchymal invasion, and branching morphogenesis in developing kidneys by mediating the signaling downstream of GDNF/RET receptor tyrosine kinases [53]. Again, this involves differentiation and chemotaxis of epithelial cells. More recently, Lindström et al. have shown that PTEN sits at the center of nephron development by integrating signaling from BMP and wnt-beta-catenin pathways [54]. Disruption of PTEN results in a loss of coordinated signaling and a loss of proper patterning of epithelial cells along the axis of the nephron tubule.

Numerous developmental effects of PTEN have been noted in the gastrointestinal tract. In the colon, PTEN (+/−) mice develop hamartomatous polyps consistent with disrupted control of differentiation of cells [55]. Further, PTEN is critical for colon homeostasis involving inflammatory repair and recovery, processes that reactivate developmental signaling mechanisms [56]. In the liver, loss of PTEN not only increases tumor susceptibility but also alters metabolism [57]. In addition, hepatomegaly is observed in mice with conditional hepatic PTEN knockout, as the ratio of liver weight to body weight increased in the mutants when compared with wild-type controls [57]. Livers harvested from mutant animals are discolored and show accumulation of triglycerides consistent with steatohepatitis associated with severe inflammation. Passive diffusion of fatty acids in hepatocytes was enhanced in PTEN null mice, and PTEN-deficient hepatocytes exhibit an increased rate of fatty acid synthesis via induced fatty acid synthase [57]. PTEN-deficient mice exhibit defects in glucose metabolism, including decreased fasting plasma glucose levels and reduced serum insulin levels [58]. Clearance of glucose was accelerated in the absence of PTEN concurrent with increased liver glycogen storage.

Pancreatic islet beta-cell specific PTEN-deficient mice exhibit increases in islet cell numbers, total islet mass, and an increase in *β*-cell progenitor proliferation during embryonic development and early postnatal life [59]. Adult PTEN mutant mice were hypoglycemic and were resistant to streptozotocin-induced diabetes. These mice are significantly smaller than controls and have a shorter lifespan. In some studies, severe hypoglycemia is associated with seizure activity and premature death before 5 weeks of age [59]. In addition PTEN (+/−) mice exhibit protection against increased insulin production during reactive oxygen species-dependent type two diabetes and insulin-resistance upon aging [60].

PTEN's role in maintaining glucose balance is not limited to the liver and pancreas but also involves adipocytes and myocytes. Inactivation of PTEN specifically in adipocytes produces no obvious gross morphological effects on the adipose tissue or adipose tissue mass. However, this mouse exhibits increased systemic glucose tolerance and insulin sensitivity complete with decreased fasting insulin and resistin levels [61]. By controlling serum insulin and resistin levels, PTEN regulates insulin sensitivity and AMP kinase activity in the liver. Similarly, skeletal and cardiac muscle cell-specific deletion causes increased systemic glucose tolerance and insulin sensitivity [62]. Interestingly, however, PTEN loss in these tissues seems to protect these mice from streptozotocin-induced diabetes and hyperglycemia indicating that the insulin hypersensitivity exhibited in tissue-selective PTEN-deficient mice allowed them to maintain proper glucose levels.

PTEN regulates proper lung development. Specific lung deletion of PTEN during embryogenesis results in impaired lung morphogenesis and early postnatal death [61]. The lungs of these neonates exhibit increased epithelial cell proliferation and decreased alveolar cell differentiation. Adults exhibit bronchiolar and alveolar epithelial hyperplasia. Mutant mice exhibit hypertrophy of alveolar cells derived from bronchioalveolar stem cells [61]. Predictably, adenocarcinoma was evident in aged adults. Further study of PTEN's role in lung development revealed that PTEN-regulated signaling mediates lung endodermal morphogenesis coordinate with the overall developmental process in the lung, and it is central to the interaction of several signaling pathways including the TGF beta-SMAD and Nkx2.1 pathways [62].

Normal development of the vascular system is also dependent on proper PTEN function. PTEN knockout in endothelial and endocardial cells in mice results in embryonic lethality due to increased capillaries and endothelial cell hypertrophy [63]. These mutant embryos exhibit pericardial cavity enlargement, leakage of blood into the pericardial cavity, and enlarged trunk vessels secondary to pericyte and vascular smooth muscle cell recruitment to blood vessels [63]. In addition, cardiomyocyte-specific deletion of PTEN leads to cardiac phenotypes via development of cardiac hypertrophy and contractile defect [64]. Future studies on PTEN will hopefully be targeted at assessing injury-related responses in the cardiovascular system, as deletion of PTEN in cardiomyocytes has been shown to protect the heart from maladaptive remodeling upon biomechanical stress.

PTEN regulates developing bone and cartilage by protecting against overgrowth of the skeletal system. Mice with deletion of PTEN in osteochondroprogenitor cells exhibit growth plate dysfunction and overgrowth of the vertebrae [65]. The bodies of these mice are notably longer than wild-type controls, demonstrating a critical defect that results in loss of proper skeletal development. Interestingly, this increase in length was not due to bone cell proliferation increases as might have been expected but rather is due to increased matrix deposition and cellular hypertrophy [65]. In a second model, osteoblast-specific knockdown of PTEN expression results in increased bone volume and density [66]. Interestingly, this mutation is associated with improved intramembranous and late endochondral fracture healing. This is now an active area of research within the bone remodeling research field.

While PTEN remains virtually synonymous with cancer biology, the studies presented here make the importance of proper PTEN signaling for regulating a wide variety of developmental processes clearly evident. From regulation of growth, differentiation, glucose maintenance, and repair and recovery to gross effects on organ and systems development, PTEN is a protein with vast consequence in the field of developmental biology, and the signaling affected by its expression is expansive. The role PTEN plays in development has clear correlates to human disease. Further, PTEN's effects on development acquire greater importance when the role that developmental signaling plays in cancer growth and progression is considered. Therefore, an extensive knowledge of how PTEN is regulated by developmental signaling, apart from somatic or genetic mutation, is required for complete understanding of PTEN's role in these important physiological processes.

## 3. PTEN Regulation as Part of Developmental Signaling

### 3.1. Intrinsic and Extrinsic PTEN Regulation

**Figure 3:**
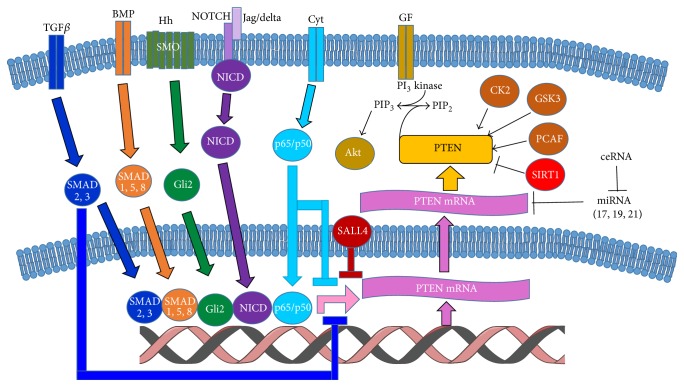
Summary of regulation mechanisms for PTEN critical for its action in developmental biology. PTEN transcription is regulated by TGF*β*, BMP, Hh, NOTCH, and cytokine signaling in cells during development. miRNAs and ceRNAs regulate PTEN message stability. Phosphorylation or acetylation by then can regulate PTEN's protein stability, enzymatic activity, and cellular localization in a positive or negative manner. All told, the regulation of PTEN is complex and precise during development, and perturbations in this paradigm have profound effects.

Most studies evaluating the role of PTEN in human diseases have been centered upon mutation in PTEN itself, and most of this work is done in its relationship to cancer initiation and progression. There is no question that mutations of PTEN play a critical role in this regard. Studies over the last decade, however, have discovered a tremendous diversity in the regulation of PTEN expression and found that PTEN is tightly controlled both transcriptionally and posttranscriptionally [67]. Recent studies to date have implicated microRNAs in PTEN suppression [68], and the enzymatic phosphatase activity of PTEN is also regulated posttranslationally via phosphorylation, ubiquitination, or oxidation [69, 70]. This section highlights the multifaceted mechanisms of PTEN regulation, supported by the rapidly expanding literature on PTEN's role in growth and patterning of tissues during development. In addition, special emphasis is given to mediators and pathways involved in development including insulin-like growth factor, transforming growth factor, bone morphogenetic proteins, NOTCH, forkhead transcription factors (Fox), and others. PTEN's complex regulation at the levels of transcription, mRNA stability, posttranslational modifications, and miRNA levels has become a critical series of mechanisms deserving a comprehensive review (for an overview, see Figure 3).

### 3.2. Developmental Signaling Regulating PTEN Expression and Action

The regulation of PTEN expression occurs at transcriptional, posttranscriptional, posttranslational, and protein-protein interaction levels [71]. PTEN's function is further controlled by its cellular location. In this section, we highlight developmental pathways involved in regulating PTEN expression at all these regulation points and make special reference to connections between signal transduction and development.

#### 3.2.1. Developmental Pathways in PTEN Gene Expression

One critical aspect of PTEN's regulation during development begins at the transcriptional level. To date, PTEN expression has been found to be regulated at the transcriptional level by pathways involving p53, PPAR*γ*, Egr-1, NF-*κ*B, and SMADs, and this regulation affects the development of proper immune function, the vasculature, the nervous system, the gastrointestinal system, and the airways [63, 72, 73]. While PTEN is constitutively expressed in many normal adult tissues, PTEN expression is altered dramatically through the course of development and in pathological settings, and a primary source of this regulation comes from bona fide developmental regulators. In addition, suppressors of cytokine signaling (SOCS) and SNAIL also induce PTEN and act by countering the growth-promoting actions of cytokines and growth factors in development and repair and recovery [74]. This mechanism has been clearly demonstrated in axonal outgrowth during neuron development and regeneration [74]. While the physiological ramifications of this regulation remain unsolved, mediators that slow or inhibit cell proliferation are often involved in inducing this protein. Developmental mediators including NOTCH, transforming growth factor beta (TGF*β*), bone morphogenetic proteins (BMPs), and hedgehogs (Hhs) all participate as players in external PTEN regulation [75–78], regulating such processes as proliferation and targeted epithelial budding and outgrowth of visceral organs [76, 77], and outgrowth in neurons [75]. As the direction of cell growth and differentiation balances on the fulcrum between the proliferative and differentiating actions of these mediators, their regulation to induce or repress PTEN expression is a critical aspect of their actions during development. The critical developmental mediator TGF*β* contains both growth promoting and inhibiting differentiation qualities, and TGF*β* induces PTEN during differentiation when growth is slowed and inhibits its expression during proliferation. In addition, the developmental regulator DJ-1 was recently identified as a novel negative regulator of PTEN by a genetic screen in* Drosophila* [79]. PTEN is also regulated through epigenetic silencing by methylation of the PTEN promoter [80, 81]. An interesting zinc-finger transcription factor, sal-like protein 4 (SALL4), has been discovered as a critical negative regulator of PTEN transcription by recruiting an epigenetic repressor complex involving an ATPase and a histone deacetylase to the PTEN promoter [80]. SALL4 plays a critical role in regulating development by modulating self-renewal in stem cells, and its function appears to at least partly involve methylation of the PTEN promoter and modulation of its transcription. Data on this mechanism are still limited, but to date this process has been shown to regulate kidney patterning during development as well as leukemogenesis [80]. Current research in the area of PTEN transcriptional regulation continues to be dynamic, and more novel regulation patterns are likely to be discovered in the near future.

#### 3.2.2. PTEN Regulation by Posttranscriptional Regulation

Recent studies have identified several interesting developmentally expressed miRNAs that regulate PTEN mRNA message stability and translatability [82]. These miRNAs typically downregulate PTEN mRNA levels by targeting it for degradation and include miR-17, miR-19, and miR-21 [83–85]. While several examples of miR-17 and miR-19-mediated PTEN RNA levels have been reported in the cancer literature, miR-21 has been described to play a critical role in repair and recovery, a developmental signaling-related process. miRNAs regulate this mechanism by modulating PTEN RNA stability and ultimately downstream Akt signaling. For example, in the pancreas, miR-21 regulates injury repair in diabetic models by targeting PTEN RNA for degradation. Inhibition of miR-21 results in autophagy in this model in a manner dependent on PTEN degradation [85]. In another example of novel miR-related PTEN regulation, the PTEN pseudogene 1 (PTENP1) acts as a decoy for PTEN-targeting miRNAs and thereby regulates PTEN expression by coding-independent activity through sequestering these miRNAs and effectively inhibiting their effects on PTEN expression [86]. Additionally, combined bioinformatic and experimental approaches have identified ceRNA transcripts that control PTEN expression by sequestering PTEN-targeting miRNAs during development of both prostate and skin [87]. Because this type of PTEN regulation, and indeed this field of gene regulation in general, is in its infancy, we are only beginning to understand the PTEN regulation by coding and noncoding ceRNAs. Still, this novel work provides a platform for further studies investigating posttranscriptional regulation patterns of PTEN expression in both developmental biology and disease conditions.

#### 3.2.3. PTEN Regulation by Posttranslational Mechanisms

The protein stability and enzyme activity of PTEN are regulated in a number of ways at the posttranslational level. First, PTEN is phosphorylated in its C-terminal tail at Ser380, Thr382, Thr383, and Ser385, and this functions to inhibit the critical PTEN phosphatase activity, thereby promoting growth in prostate and kidney cells [88]. However, these phosphorylations also stabilize the PTEN protein by dissuading ubiquitination. While this may seem like a cellular disconnect, this paradoxical phenomenon is explained by the finding that this closed, more stable PTEN conformation is prevented from associating with membranes and therefore is geographically separated from its substrates [88]. PTEN can also be activated by phosphorylation; in particular, the C2 domain of PTEN is phosphorylated at Ser229 and Thr321 by RHOA-associated protein kinase (ROCK) [89], a phosphorylation that results in its association with membranes and phosphoinositide substrates, affecting differentiation in leukocytes. PTEN is further phosphorylated at Tyr336 by the Tyr protein kinase RAK, and this phosphorylation results in its stabilization [90]. Loss of this activity has the effect of promoting breast cell proliferation and migration. Finally, PTEN is also phosphorylated at Ser370 and Thr366 by CK2 and GSK3*β* [91, 92], respectively; however, the consequences of this phosphorylation are unclear. The likely extensive signaling networks that regulate these widespread phosphorylations in PTEN are not fully studied, but the TGF/BMP-SMAD developmental pathways are known to regulate PTEN phosphorylation [93, 94]. Given the widespread application of SMAD signaling to developmental biology, the presence of SMAD-regulated PTEN phosphorylation is a critical aspect of developmental regulation in cells.

PTEN enzymatic activity is also regulated posttranslationally by acetylation [70, 95–97]. PTEN is acetylated at Lys125–Lys128 by p300/CREB-binding protein- (CBP-) associated factor (PCAF) and at Lys402 by CBP itself [95]. Acetylation at these residues inhibits PTEN's function as a phosphatase and has been shown to promote the growth of prostate epithelial cells and glial cells [95]. In addition, the deacetylase Sirtuin 1 (SIRT1) is involved in excluding PTEN from the nucleus and therefore preventing its newly characterized nuclear functions [96].

#### 3.2.4. PTEN Regulation by Protein-Protein Interactions

The conformation, stability, and subcellular distribution of PTEN is regulated by critical interactions with other proteins within cells, and these proteins are known to be critical for proper development of a number of organ systems. The guanylate kinase inverted 2 (MAGI2) and *β*-arrestins cooperate to enhance PTEN activity [98, 99]. MAGI2 phosphorylates PTEN on threonine residues at positions 382 and 383 of PTEN's carboxy terminus, allowing recruitment to cell-cell junctions [98]. Mutations to these residues prevent PTEN recruitment. Upon recruitment, *β*-arrestins modulate PTEN activity by direct binding to the C2 domain and modulating its protein phosphatase activity, including that at the level of small GTPases including RhoA/ROCK [99]. In addition, Myosin V regulates PTEN's movement to and from the plasma membrane, and this function is required for PTEN to access its phosphoinositide substrates [100] and for disrupting the proper regulation of neuronal cell size. The p85 regulatory subunit of the PI_3_ kinase enzyme itself interacts with PTEN in a novel mechanism of repressing the PI_3_K–AKT pathway [101] independent of PTEN's action as a phosphatase. It is therefore proposed that p85 regulates the PI_3_K–AKT pathway bidirectionally both through its binding to catalytic p110 subunit resulting in the generation of phosphoinositides and by binding PTEN, leading to repression. This work proposes a novel signaling model for PTEN-p85 association, in which p85*α* preferentially binds and stabilizes p110 preferentially when p85*α* levels are low, but excess p85*α* binds to and positively regulates PTEN phosphatase activity when p110 is saturated. Therefore, sustained activation of PI3K signaling and activated p110-p85 complexing is modulated homeostatically by this mechanism.

A fascinating group of recent studies have utilized proteomic library screens to identify further novel PTEN interacting proteins. First, the Na^+^/H^+^ exchanger regulatory factor (NHERF) binds to and recruits PTEN to the platelet-derived growth factor receptor (PDGFR). This represents another action that restricts PI_3_K–AKT pathway activation, independent of phosphatase activity [102]. This has the effect of inhibiting PDGF-induced cytoskeletal rearrangements and chemotactic migration of developing neurons. In addition, the mammalian disks large homologue 1 (DLG1), MAGI-2, and MAST205 proteins directly interact with PTEN and enhance PTEN stability and activity [103]. Further, neuregulin 1 (NRG1) has been shown to interact with both DLG1 and PTEN in a stable complex that has the effect of regulating myelin sheath thickness in developing mouse sciatic nerves [104]. Additional but less-characterized protein-protein interactions that modulate PTEN function include the PIP_3_-dependent RAC exchanger factor 2a (PREX2a) [105], shank-interacting protein-like 1 (SIPL1) [106], and *α*-mannosidase 2C1 (MAN2C1) [107].

#### 3.2.5. PTEN-Controlled Signal Transduction during Development

PTEN is critically important in regulating signaling pathways involved in cell growth and animal development. Through its well-characterized function as a phosphatase of PIP_3_, PTEN modulates a network of mitogen-activated signals transduced through PI_3_K–Akt signaling. This primary action of PTEN has tremendous impact on cell growth, cell migration, cell death, and cell differentiation—cellular processes that are all hallmarks of proper growth and patterning during development. The active phosphorylated PIP_3_ activates several signaling molecules including the phosphatidylinositol-dependent kinases (PDKs), followed by Akt, S6 kinase, and mTOR. Beyond this, AKT's targets include the apoptotic factor BAD, activator and executioner caspases, glycogen synthase kinase-3 (GSK-3), MDM2, p21 and p27, forkhead transcription factors (FOXOs), and nuclear receptors including the androgen and estrogen receptors [108]. Akt-mediated phosphorylation of these molecules causes activation, translocation, and increased stability.

The PTEN/PI_3_ kinase/AKT signaling cascade crosstalks to several key signal transduction pathways that are critical to developmental biology. This includes the abovementioned TGF-*β*/SMAD pathway and the Wnt/*β*-catenin pathway [109]. The TGF-*β*/SMAD pathway is one of the most seminal pathways in developmental biology, and PTEN modulates TGF*β*/SMAD signaling by reversing Akt-mediated phosphorylation of SMAD3, the critical signaling intermediate of TGF*β*/SMAD signaling [110]. Similarly, the Wnt signaling pathway is indispensable for properly regulated development, cellular proliferation, and differentiation [111]. PTEN regulates this pathway by reversing AKT kinase phosphorylation and inhibition of GSK-3*β*, resulting in *β*-catenin nuclear translocation and activation. Finally, recent studies demonstrate that PTEN regulates the expression and action of the homeobox genes NKX3.1 and hepatic nuclear factor [112], proteins that are critical to organ development.

#### 3.2.6. PTEN-Regulated Stem Cell Activities

The scientific literature in the field of tissue stem or stem-like “progenitor” cells and their role in tissue development, growth, recovery and repair has become beyond extensive in recent years. Expansion of stem/progenitor cells is a hallmark of growth and patterning during development, and regulation of these cells, including their proliferation, maintenance, and differentiation, is a critical factor of developmental regulation. Because of this, a review of the current literature on PTEN's role in stem and tissue progenitor cells is warranted in this review. PTEN plays an important role in regulating the homeostasis of stem/progenitor cells in multiple tissues. Interestingly, PTEN regulates both self-renewal and proliferation, but paradoxically it also plays a role in stem cell exhaustion. For example, the FOXO family of transcription factors controls stem cell proliferation and survival in hematopoietic stem cells [113], and PTEN signaling is a critical modulator of FOXO activity. FOXO3A-deficient HSCs exhibit increased proliferation rate, decreased quiescence, and a reduced ability to repopulate bone marrow after transplantation into recipient mice. Additionally, the previously mentioned Wnt/*β*-catenin signaling pathway is critical for HSC self-renewal, and its activation is well known to be modulated by PTEN. Interestingly, PTEN loss and AKT activation in HSCs lead to a moderate increase in the level of unphosphorylated *β*-catenin, but this is not sufficient to maintain* Pten*-null HSC self-renewal [114]. Finally, NOTCH signaling plays a key role in regulating neural stem cell expansion [115] and, as stated previously, NOTCH pathway activation leads to reduced PTEN expression in neural and hematopoietic stem cells.

## 4. Conclusions

PTEN acts as the key endogenous negative modulator of phosphoinositide signaling in mammalian cells, and therefore its role in normal cell homeostasis, proliferation, apoptosis, and many additional components of cell biology is extensive. Because PTEN regulates cancer progression, much of what has been studied regarding PTEN relates to tumorigenesis and tumor growth. While this is warranted because of the relevance of cancer to human health, PTEN has substantial but underappreciated effects in normal tissue and organ development, immune system development and regulation, axonal regeneration, glucose regulation, and benign growth disorders. PTEN is often dogmatically thought of as a constitutive protein that exhibits lost activity during diseases, but what is underappreciated is that PTEN has a very complex regulation network that involves signaling pathways that regulate PTEN transcription, transcript stability, posttranslational modifications, protein-protein interactions, and cellular location. PTEN then in turn regulates numerous developmental signaling networks that are critical to development and adult tissue maintenance.

## Abbreviations

Bcl2: B-cell lymphoma 2
Bim: BCL-2-interacting mediator
CK2: Casein Kinase 2
CREB: Cyclic AMP response element binding protein
DLG: Disks large homolog 1
Egr-1: Early growth response protein 1
Erk: Extracellular regulated kinase
FOX: Forkhead transcription factors
GSK3*β*: Glycogen synthase kinase 3
JNK: Jun-terminal kinase
MAGI2: Membrane-associated guanylate kinase inverted 2
MAN2C1: Mannosidase, alpha, class 2C, member 1
MDM2: Mouse double minute 2 homolog
mTOR: Mechanistic target of rapamycin
NHERF: Sodium-hydrogen antiporter 3 regulator 1
NRG1: Neuregulin 1
PCAF: P300/CBP-associated factor
PDZ: Postsynaptic density protein—Disc large tumor suppressor—Zonula occludens-1 protein
PIP3: Phosphoinositide triphosphate
PPAR*γ*: Peroxisome proliferating antigen receptor
PREX2a: Phosphatidylinositol-3,4,5-trisphosphate-dependent Rac exchange factor A
PTEN: Phosphatase and Tensin homologue
ROCK: Rho-associated, coiled-coil-containing protein kinase
SALL: Spalt-like transcription factor-1
SIPL1: Shank-interacting protein-like 1
SIRT1: Sirtuin 1
SMAD: Small mothers against decapentaplegic
SOCS: Suppressor of cytokine signaling
TGF*β*: Transforming growth factor-beta.
